# Supplementary material for: A Disintegrin and Metalloproteinase 9 (ADAM9) in Advanced Hepatocellular Carcinoma and Their Role as a Biomarker During Hepatocellular Carcinoma Immunotherapy
Source: Cancers (Basel). 2020 Mar 21;12(3):745. doi: 10.3390/cancers12030745 (PMC7140088; doi:10.3390/cancers12030745)
Supplement: Supplementary file 1 [file cancers-12-00745-s001.pdf]

Supplementary Appendix for

A Disintegrin And Metalloproteinase 9 (ADAM9) in Advanced Hepatocellular Carcinoma (HCC) and Its Role as A Biomarker during HCC Immunotherapy

Coauthored by Sooyeon Oh, YoungJoon Park, Hyun-Jung Lee, Jooho Lee*, Soo-Hyeon Lee, Young-Seok Baek, Su-Kyung Chun, Seung-Min Lee, Mina Kim, Young-Eun Chon, Yeon-Jung Ha, Yuri Cho, Gi-Jin Kim, Seong-Gyu Hwang, KyuBum Kwack

* Correspondence: ljh0505@cha.ac.kr; Tel.: +82-31-780-1811

Table S1. Baseline patient and tumor characteristics of the study population (n = 10)

Variables	(Mean ± SD or No. [%])
Age (years)	57.4 ± 18.6
Male gender	8 (80%)
Platelet count ($x10^3/mm^3$)	162.0 ± 114
ALT (IU/L)	33.7 ± 48.3
Total bilirubin (mg/dL)	1.77 ± 4.03
Albumin (g/dL)	3.75 ± 0.75
PT INR	1.24 ± 0.14
Underlying disease (HBV/HCV/Non-viral)	8 (80%) / 1 (10%) / 1 (10%)
PIVKA II (mAU/mL)	17831.6 ± 57168.4
Serum AFP (ng/ml)	30908 ± 30851.4
Child-Pugh class (A/B/C)	7 (70%) / 3 (30%) / 0 (0%)
Tumor size (cm)*	8.36 (3.0 – 22.0)
Modified UICC stage (III/IV)	1 (10%) / 9 (90%)

*Median and range. Abbreviations: SD, standard deviation; No, number; ALT, alanine transaminase; PT INR; prothrombin time international normalized ratio; HBV, hepatitis B virus; HCV, hepatitis C virus; AFP, alpha-fetoprotein; UICC, Union for International Cancer Control

Table S2. Changes in the proportions of immune checkpoint molecules and immune cells in the HCC patient (Subject #6), who showed complete tumor response following regorafenib and NK cell combination therapy. Immunophenotyping was done when the patient reached PR in October 2018 and CR in December 2019.

Cell type	Checkpoint molecule	Phenotype marker	PR, Oct 2018	CR, Dec 2019
T cells (%)		$CD3^{+}$	76.37	42.57
Helper T cells (%)		$CD3^{+}CD4^{+}$	51.25	24.29
Cytotoxic T cells (%)		$CD3^{+}CD8^{+}$	24.78	14.37
B cells (%)		$CD19^{+}$	6.68	12.09
NK cells (%)		$CD3^{-}CD56^{+}$	14.81	43.72
NKT cells (%)		$CD3^{+}CD56^{+}$	6.01	2.26
Cytotoxic T cells	PD-1 (%)	$CD3^{+}CD8^{+}PD\text{-}1^{+}$	41.21	23.6
	TIM-3 (%)	$CD3^{+}CD8^{+}TIM3^{+}$	8.89	21.59
	LAG-3 (%)	$CD3^{+}CD8^{+}LAG3^{+}$	18.33	26.84
	BTLA (%)	$CD3^{+}CD8^{+}BTLA^{+}$	59.27	62.67
Helper T cells	PD-1 (%)	$CD3^{+}CD4^{+}PD\text{-}1^{+}$	34.41	41.17
	TIM-3 (%)	$CD3^{+}CD4^{+}TIM3^{+}$	21.26	30.79
	LAG-3 (%)	$CD3^{+}CD4^{+}LAG3^{+}$	39.33	43.8
	BTLA (%)	$CD3^{+}CD4^{+}BTLA^{+}$	34.7	70.36
NK cells	PD-1 (%)	$CD3^{-}CD56^{+}PD\text{-}1^{+}$	1.35	3.76
	TIM-3 (%)	$CD3^{-}CD56^{+}TIM\text{-}3^{+}$	24.18	1.02
	LAG-3 (%)	$CD3^{-}CD56^{+}LAG3^{+}$	12.39	11.7
	BTLA (%)	$CD3^{-}CD56^{+}BTLA^{+}$	6.68	1.39
	CD16 (%)	$CD3^{-}CD56^{+}CD16^{+}$	98.31	99.54
	NKG2D (%)	$CD3^{-}CD56^{+}NKG2D^{+}$	81.5	82.17

Abbreviations: HCC, hepatocellular carcinoma; NK, natural killer; NKT, natural killer-T; PD-1, programmed cell death 1 protein; TIM-3, T cell immunoglobulin- and mucin-domain-containing molecule 3; LAG-3, lymphocyte activation gene-3; BTLA, B and T lymphocyte attenuator; NKG2D, natural killer group 2D; PR, partial response; CR, complete response; Oct, October; Dec, December.

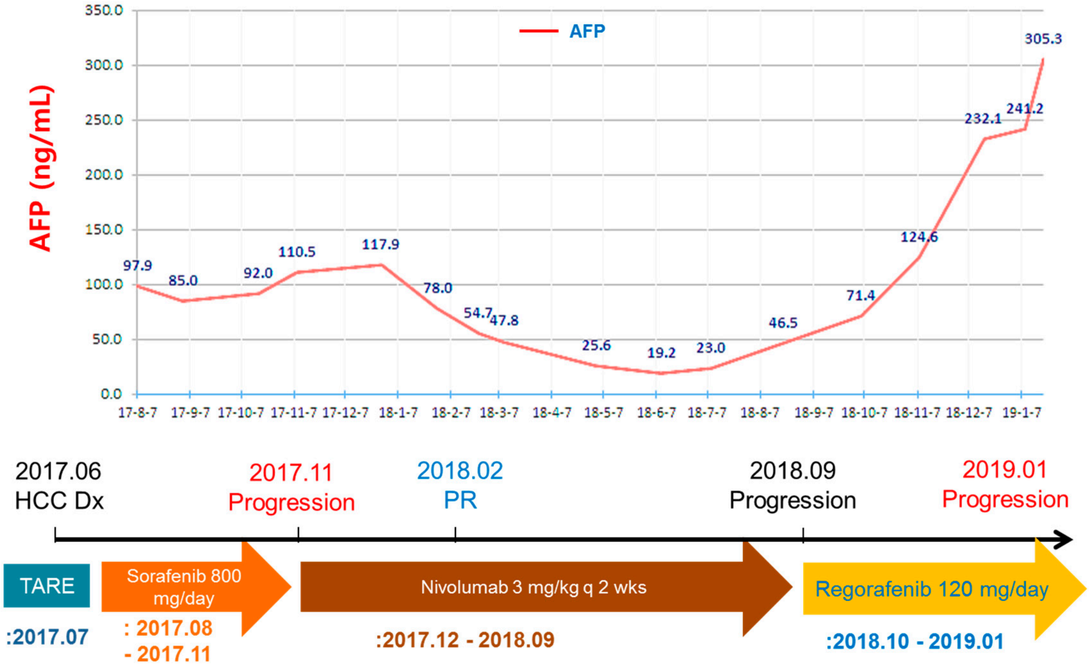

Figure S1. Clinical course and serum AFP levels of Subject #9.

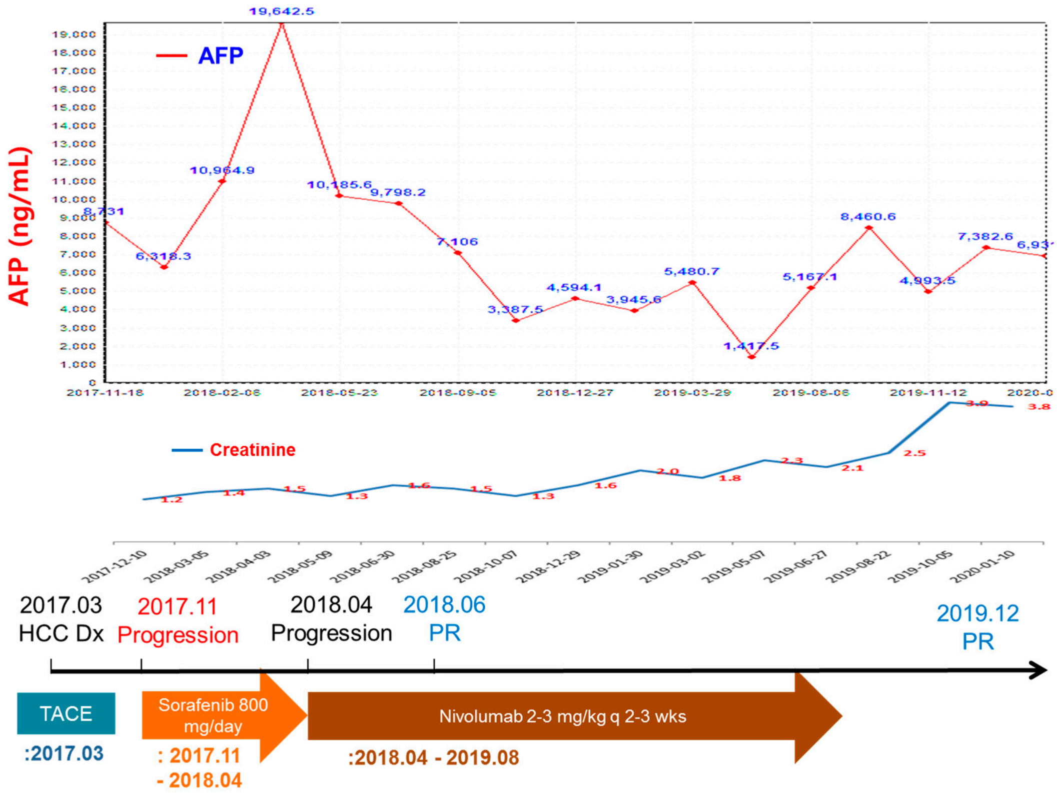

Figure S2. Clinical course and serum AFP levels of Subject #10.

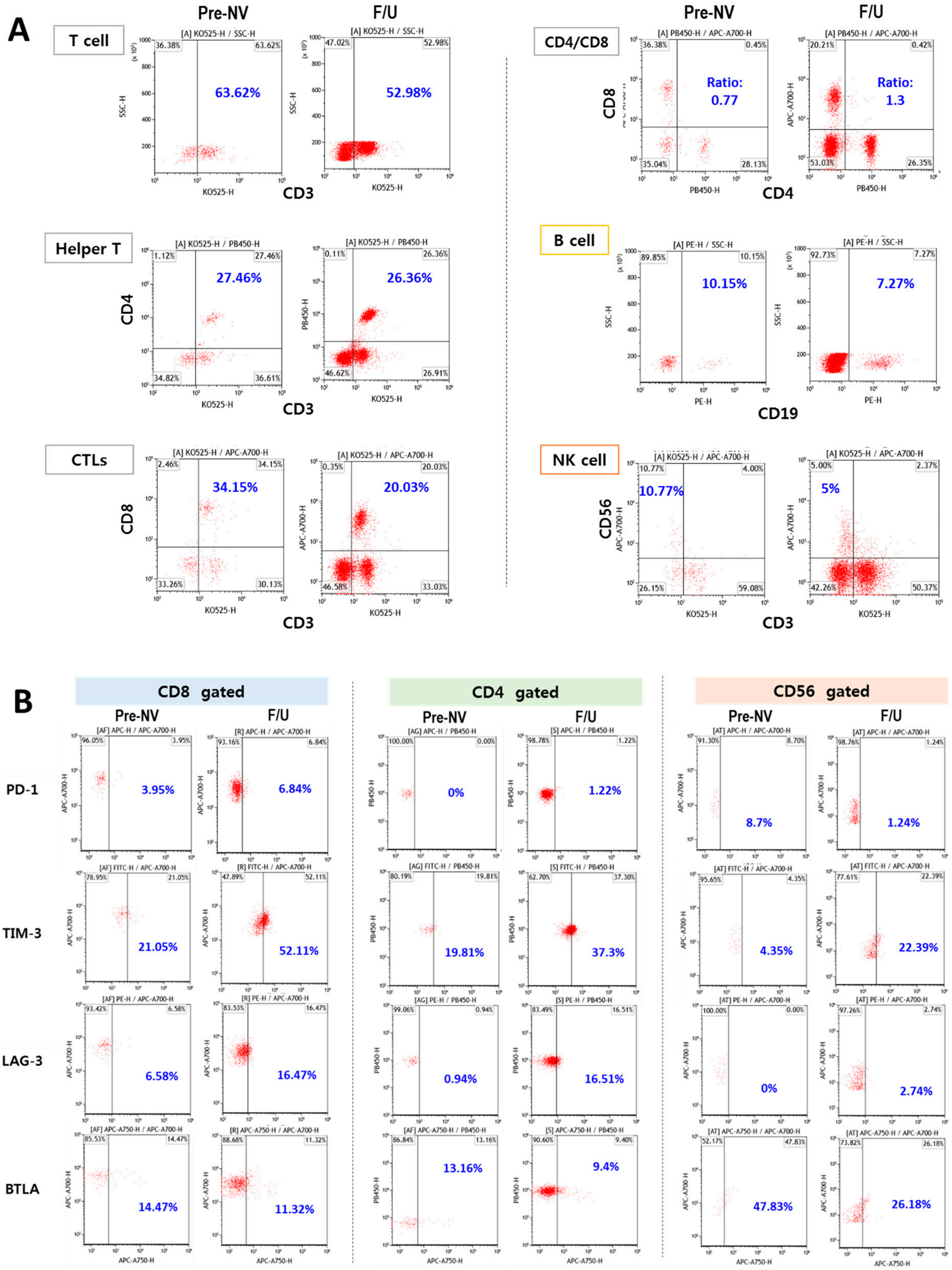

Figure S3. Lymphocyte distribution (A) and inhibitory checkpoint molecule expression (B) before and after nivolumab therapy in Subject #7. Abbreviations: NV, nivolumab; F/U, follow-up; CD, cluster differentiation; CTL, cytotoxic T lymphocytes; NK, natural killer; PD-1, programmed cell death 1 protein; TIM-3, T cell immunoglobulin- and mucin-domain-containing molecule 3; LAG-3, lymphocyte activation gene-3; BTLA, B and T lymphocyte attenuator.

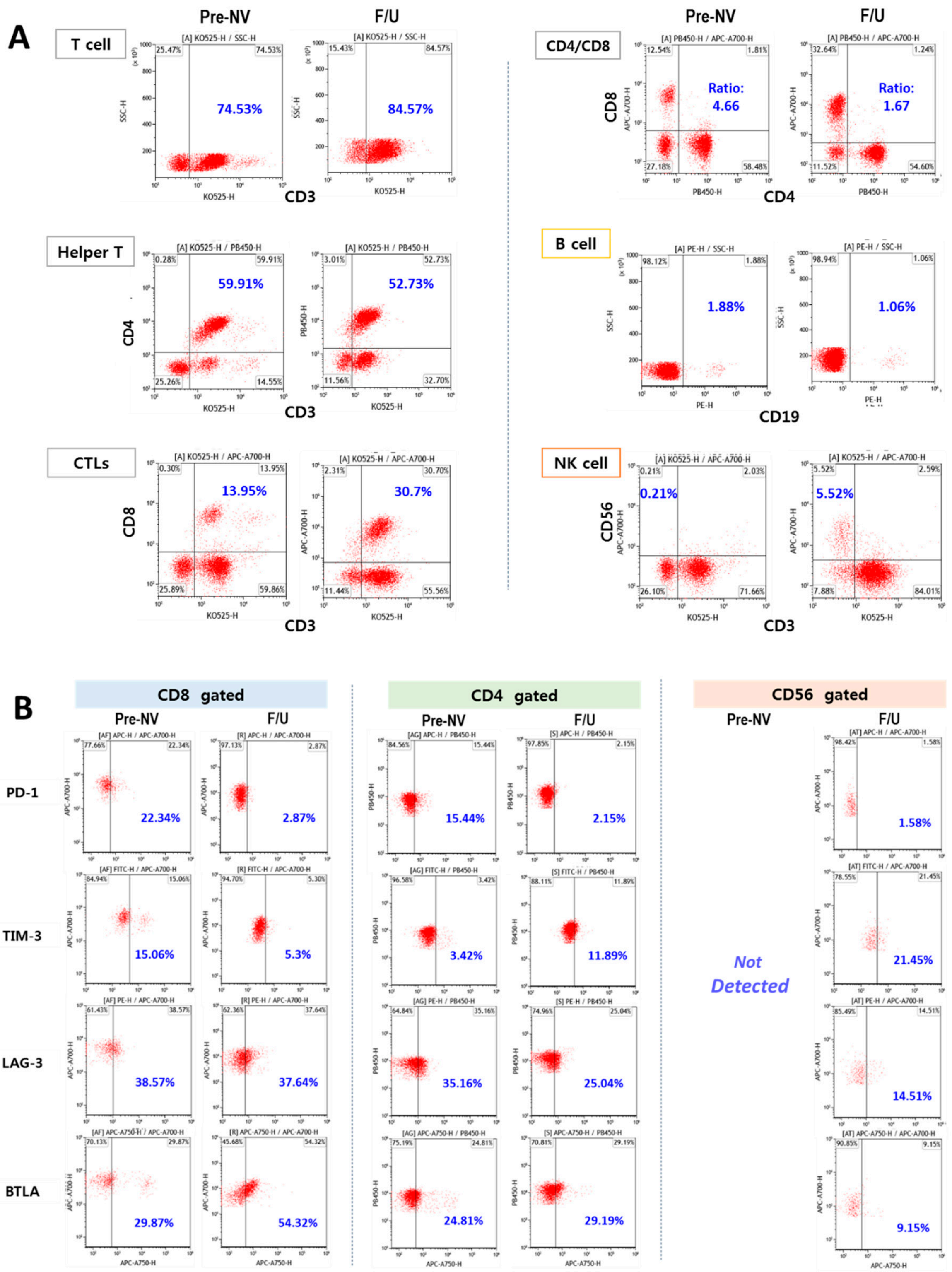

Figure S4. Lymphocyte distribution (A) and inhibitory checkpoint molecule expression (B) before and after nivolumab therapy in Subject #8. Abbreviations: NV, nivolumab; F/U, follow-up; CD, cluster differentiation; CTL, cytotoxic T lymphocytes; NK, natural killer; PD-1, programmed cell death 1 protein; TIM-3, T cell immunoglobulin- and mucin-domain-containing molecule 3; LAG-3, lymphocyte activation gene-3; BTLA, B and T lymphocyte attenuator.

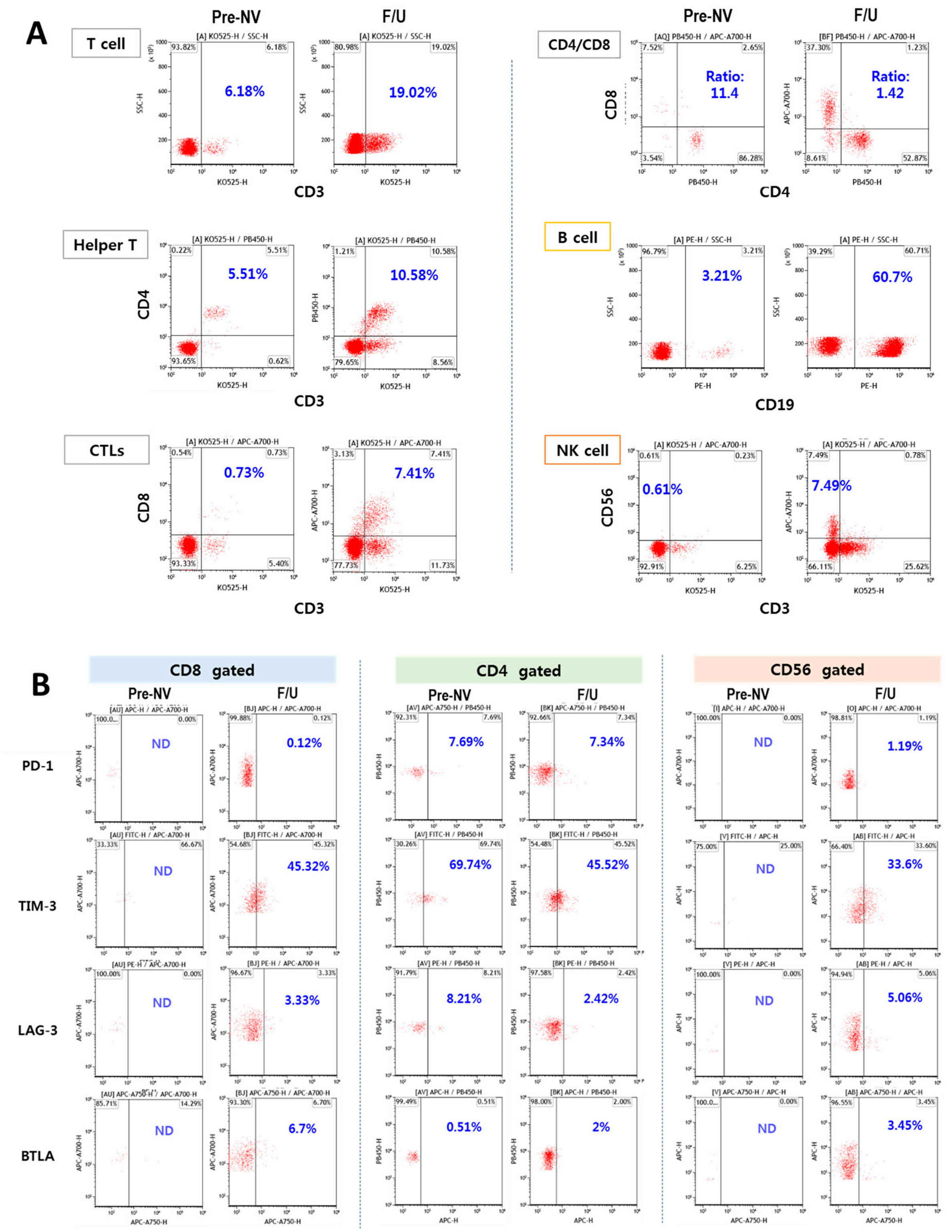

Figure S5. Lymphocyte distribution (A) and inhibitory checkpoint molecule expression (B) before and after nivolumab therapy in Subject #9. Abbreviations: NV, nivolumab; F/U, follow-up; CD, cluster differentiation; CTL, cytotoxic T lymphocytes; NK, natural killer; PD-1, programmed cell death 1 protein; TIM-3, T cell immunoglobulin- and mucin-domain-containing molecule 3; LAG-3, lymphocyte activation gene-3; BTLA, B and T lymphocyte attenuator; ND, not detected.

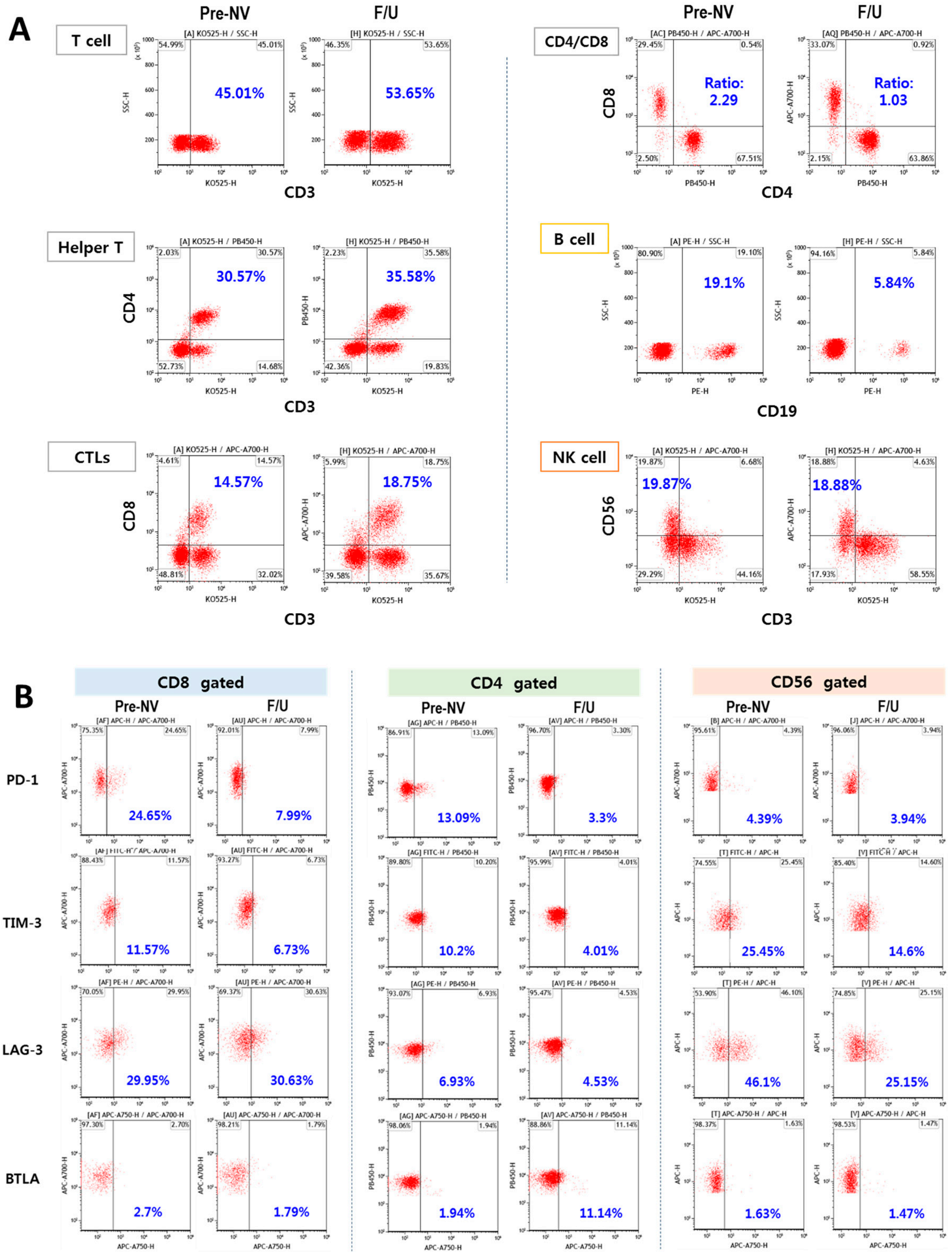

Figure S6. Lymphocyte distribution (A) and inhibitory checkpoint molecule expression (B) before and after nivolumab therapy in Subject #10. Abbreviations: NV, nivolumab; F/U, follow-up; CD, cluster differentiation; CTL, cytotoxic T lymphocytes; NK, natural killer; PD-1, programmed cell death 1 protein; TIM-3, T cell immunoglobulin- and mucin-domain-containing molecule 3; LAG-3, lymphocyte activation gene-3; BTLA, B and T lymphocyte attenuator.

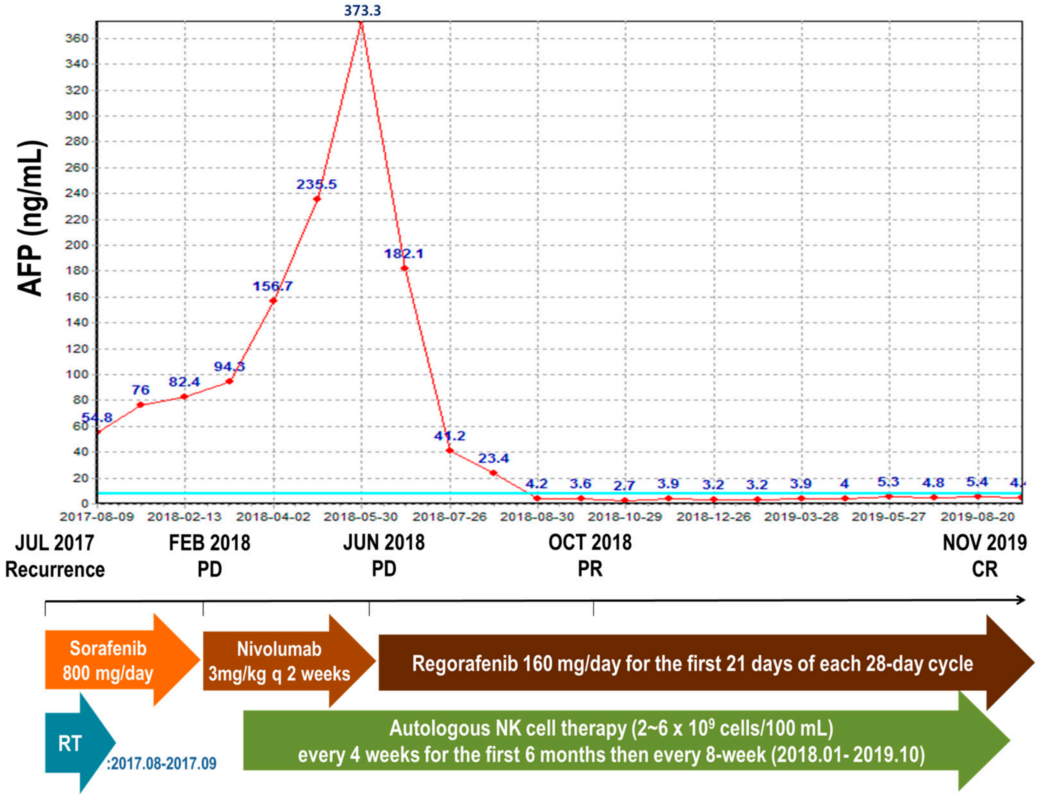

Figure S7. Clinical course and serum AFP level of Subject #6. This 59-year-old man with CHB was diagnosed with intrahepatic HCC at the segment 4 of liver (Supplementary Figure S8 (A)). The tumor had been taken out with surgical resection in March 2017. In 5 months, however, intrahepatic recurrence was noted with malignant thrombi at the right portal vein. Sorafenib in combination with radiotherapy (40 Gy in equivalent dose of 2 Gy per fraction for target lesion including portal vein tumor thrombi) was introduced from August 2017. After 6 months with sorafenib administration, the HCC progressed further (Supplementary Figure S8 (B)) with marked elevation of serum alpha-fetoprotein (AFP) levels (Figure S7). Therefore, nivolumab was started as the second-line chemotherapy. To enhance the therapeutic efficacy of nivolumab, activated autologous NK cell therapy (2~6 x 10^9 cells/100 mL, intravenous infusion every 4 weeks) was combined since January 2018. During the first 3 months of nivolumab and NK cell combination therapy, a follow-up CT scan showed no interval change in tumor size. However, the serum AFP levels increased continuously. Suspecting disease progression in molecular level, nivolumab was switched to the third-line chemotherapy, regorafenib. To allow synergistic action of NK cells upon suppression of ADAM9 protease and sMICA by regorafenib, the patient continued receiving the NK cell therapy every 4 weeks up to 6 times and then every 8 weeks up to total 15 times. The serum AFP levels started to decrease with the administration of regorafenib. In 3 months (September 2018), tumor regression was confirmed in a follow-up CT scan, and serum AFP levels reached a normal value (Supplementary Figure S8 (C)). In December 2018 (6 months after regorafenib with concomitant NK cell therapy), the patient achieved near complete regression of HCC (Figure S7). At this point, we acquired his blood sample for this study, and ADAM9 mRNA was not detected at all in his plasma (Figure 1). The last follow-up CT scan was done in November 2019 and revealed a CR of HCC (Supplementary Figure S8 (D)). In the meantime, the serum AFP level continued to be in the normal range. The serum protein induced by vitamin K absence or antagonist-II (PIVKA-II) levels also showed the same pattern change as the serum AFP levels (data not shown).

Abbreviations: AFP, alpha-fetoprotein; CHB, chronic hepatitis B; HCC, hepatocellular carcinoma; PD, progressive disease; PR, partial response; CR, complete response; CT, computed tomography; RT, radiotherapy; NK, natural killer.

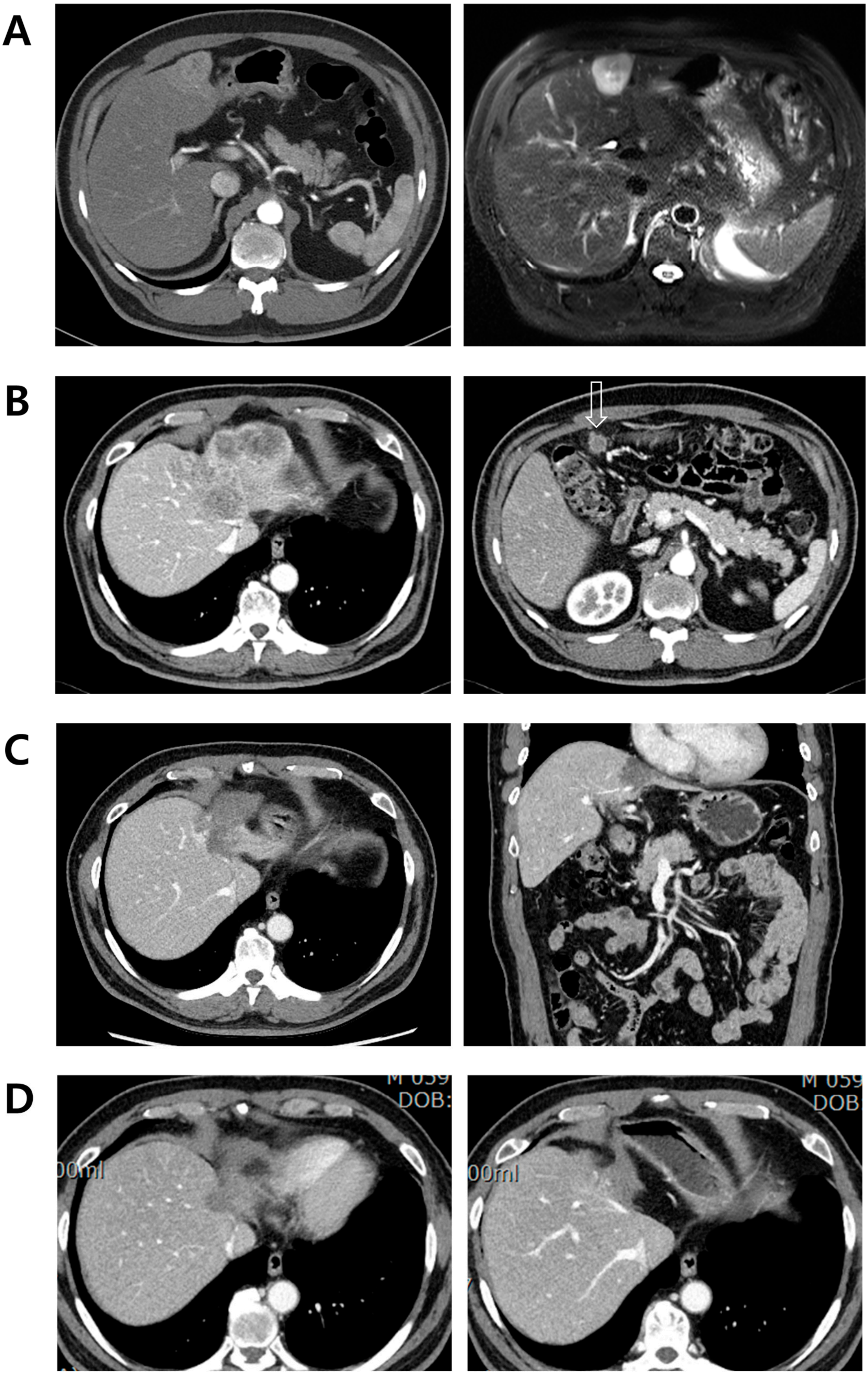

Figure S8. Completely resolved HCC after regorafenib and autologous NK cell combined therapy in one patient (Subject #6). (A) About 3.5 cm arterial enhancing mass at hepatic segment 4 was noted on liver dynamic CT (left) and MRI (right) taken at March 2017 before surgical resection. In 5 months,

however, intrahepatic recurrence was noted with malignant thrombi at the right portal vein. Sorafenib in combination with radiotherapy (40 Gy in equivalent dose of 2 Gy per fraction for target lesion including portal vein tumor thrombi) was introduced from August 2017. Sorafenib was administered for intrahepatic recurrence since August 2017. (B) In 6 months (January 2018), intrahepatic recurrence was noted (left) with malignant thrombi at the right portal vein and a tumor seeding nodule newly appeared in gastrohepatic ligament (right, arrow). As the second-line chemotherapy, nivolumab was started, and autologous NK cell therapy was combined. In 3 months, HCC showed no interval change in tumor size, however, the serum AFP level continuously increased (Figure 6). Suspecting disease progression in molecular level, nivolumab was switched to third-line chemotherapy, regorafenib, and the NK cell therapy was continued. (C) Three months after regorafenib (September 2018), tumor regression was noted in a follow-up CT scan with a decrease of serum AFP level. (D) The follow-up CT scan in November 2019 showed complete regression of HCC and serum AFP level was within normal range (Figure 7). Abbreviations: HCC, hepatocellular carcinoma; NK, natural killer; CT, computed tomography; MRI, magnetic resonance imaging; AFP; alphafetoprotein.

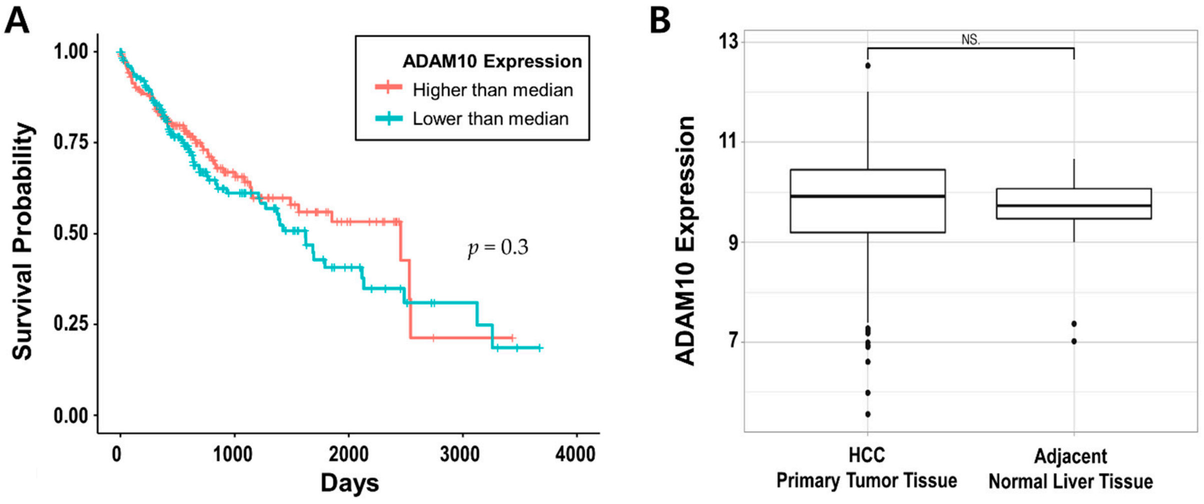

Figure S9. Effect of *ADAM10* expression on HCC prognosis in the TCGA database. (A) Kaplan–Meier plot of HCC patients (n=370) according to *ADAM10* expression levels higher or lower than median (n=185 for each group). (B) Box-plot comparing *ADAM10* expression between HCC primary tumor (n=370) and adjacent normal liver tissue (n=50).
Abbreviations: HCC, hepatocellular carcinoma; TCGA, The Cancer Genome Atlas

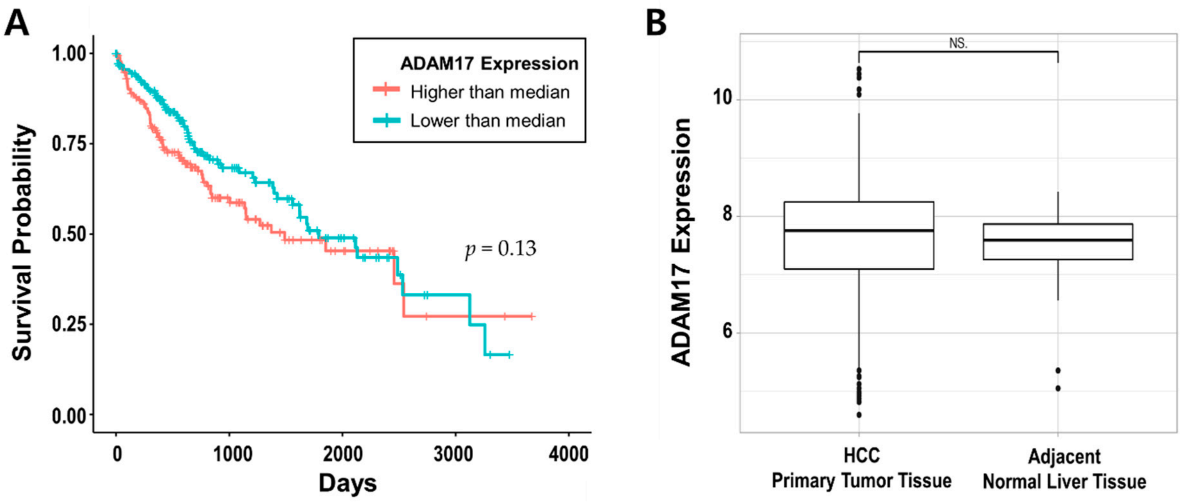

Figure S10. Effect of *ADAM17* expression on HCC prognosis in the TCGA database. (A) Kaplan–Meier plot of HCC patients (n=370) according to *ADAM17* expression levels higher or lower than median (n=185 for each group). (B) Box-plot comparing *ADAM17* expression between HCC primary tumor (n=370) and adjacent normal liver tissue (n=50).
Abbreviations: HCC, hepatocellular carcinoma; TCGA, The Cancer Genome Atlas
